# Complement Receptors C5aR and C5L2 Are Associated with Metabolic Profile, Sex Hormones, and Liver Enzymes in Obese Women Pre- and Postbariatric Surgery

**DOI:** 10.1155/2014/383102

**Published:** 2014-03-26

**Authors:** Reza Rezvani, Jessica Smith, Marc Lapointe, Picard Marceau, Andre Tchernof, Katherine Cianflone

**Affiliations:** Centre de Recherche de l'Institut Universitaire de Cardiologie & Pneumologie de Québec, Université Laval, Y4332, 2725 Chemin Ste-Foy, Québec, QC, Canada G1V 4G5

## Abstract

*Objective*. Obesity is associated with metabolic dysfunction with sex differences and chronic, low-grade inflammation. We proposed that hepatic expression of immune complement C3 related receptors (C3aR, C5aR, and C5L2) would be associated with pre- or postmenopausal status and metabolic profile in severely obese women. We hypothesized that C5L2/C5aR ratio, potentially influencing the ASP/C5L2 metabolic versus C5a/C5aR immune response, would predict metabolic profiles after weight loss surgery. * Materials and Methods*. Fasting plasma (hormone, lipid, and enzyme analysis) and liver biopsies (RT-PCR gene expression) were obtained from 91 women during surgery. * Results*. Hepatic C5L2 mRNA expression was elevated in pre- versus postmenopausal women (*P* < 0.01) and correlated positively with circulating estradiol, estrone, ApoB, ApoA1, ApoA1/B, waist circumference, age, and LDL-C (all *P* < 0.05). While plasma ASP was lower in pre- versus postmenopausal women (*P* < 0.01), the hepatic C5L2/C5aR mRNA ratio was increased (*P* < 0.001) and correlated positively with estrone (*P* < 0.01) and estradiol (*P* < 0.001) and negatively with circulating ApoB and liver enzymes ALT, AST, and GGT (all *P* < 0.05). Over 12 months postoperatively, liver enzymes in low C5L2/C5aR mRNA ratio group remained higher (ALP and ALT, *P* < 0.05, AST and GGT, *P* < 0.001 2-way-ANOVA). * Conclusion*. C5L2-C5aR association with other mediators including estrogens may contribute to hepatic metabolic and inflammatory function.

## 1. Introduction

Obesity is associated with increased morbidity and mortality from cardiovascular disease, type 2 diabetes, and fatty liver disease, all of which have been clearly linked to a chronic, low-grade inflammatory status. Multiorgan involvement of obesity-induced inflammation (liver and adipose tissue), sex differences in obesity and obesity-related conditions (including pre- versus postmenopausal status), and dynamic interactions between immune and metabolic responses (termed metaflammation) [1] are all considered important determinants of metabolic disease in obesity.

The complement system is recognized as a key immune regulatory system for cell and tissue homeostasis [2]. Complement component C3 plays a central role in the activation of the complement system [3]. One major source of circulating complement proteins, such as C3, is the liver [4], although adipose tissue and macrophages also secrete C3 [5]. The liver is constantly exposed to complement-activating pathogens via the portal venous system [6]. Proximal and distal activation of complement C3 leads to production of anaphylatoxins C3a and C5a, respectively, which have multiple immune functions including stimulation of histamine secretion and oxidative burst and chemotactic activity as well as secretion of various cytokines (reviewed in [7]). In recent years, C3a and C5a have also been found to have multiple metabolic functions in tissue homeostasis [8–10] and tissue regeneration [11] as well as brain development [12].

In circulation, C5a and C3a are rapidly cleaved by carboxypeptidases to generate C5adesArg and C3adesArg, respectively. C3adesArg, also known as acylation stimulating protein (ASP), is a lipogenic hormone, involved in lipid storage and energy homeostasis (reviewed in [13]). ASP stimulates free fatty acid incorporation into adipose tissue by increasing triglyceride synthesis and glucose uptake and reducing triglyceride lipolysis in adipocytes [13]. These complement components bind to a family of three receptors, which belong to the superfamily of G-protein-coupled receptors: the C3a receptor (C3aR), C5a receptor (C5aR), and C5a receptor-like 2 (C5L2). All three receptors (C3aR, C5aR, and C5L2) have demonstrated roles in the immune process with more recent data in knockout (KO) mice demonstrating emerging roles in energy metabolism [8, 10, 13, 14].

C3aR, which binds C3a, has demonstrated roles in asthma, sepsis, and liver regeneration as well as neuron maturation [12]. Further, based on studies in C3aR knockout mice, C3aR plays a role in insulin resistance and adipose tissue macrophage infiltration [9].

C5aR, which binds C5a and, to a lesser extent, C5adesArg, is involved in many inflammatory diseases including asthma, sepsis, rheumatoid arthritis, and inflammatory bowel diseases as well as cancer and liver diseases [7]. One previous study showed that C5a stimulates food intake after central administration [15], while a very recent study in C5aR knockout mice demonstrated decreased body weight and fat storage regardless of diet (low fat chow or diet-induced obesity regimen) [8].

C5L2 binds C5a as well as C5adesArg, the latter with a higher affinity than to C5aR [16]. C5L2 has been postulated to be both a nonsignaling decoy receptor for C5a [17, 18] as well as a signaling receptor [19]. In previous studies, we have demonstrated a role for C5L2 in ASP function [20, 21], with binding of ASP/C3adesArg and C3a to C5L2 [16, 20], downstream signaling activation [19, 22], and functional output (such as increased glucose uptake and TG synthesis) [23, 24]; however, the binding of ASP/C3adesArg to C5L2 remains controversial [25]. Recent studies have demonstrated formation of C5L2/C5aR homo- and heterodimers, with colocalization upon stimulation with either ASP/C3adesArg or C5a [26], as confirmed recently [27].

Sex steroids are an additional factor impacting body fat distribution patterns, circulating lipids, and prevalence of metabolic diseases. Recent studies have shown that 17*β*-estradiol may play a role in reducing the inflammatory response in adipose tissue as well as the cardiovascular and neural systems [28–30]. Further, various cell studies have indicated that sex hormones may play a role in the regulation of C3 [31], C5aR [32, 33], and the C5L2/ASP response [34].

In relation to liver function specifically, a recent study in humans indicated that plasma C3a was associated with liver steatosis and hepatocellular injury in individuals consuming considerable amounts of alcohol daily as well as in severely obese people [35]. C3KO mice and C5L2KO mice on a high-fat diet are prone to develop enhanced hepatic steatosis as a result of increased hepatic triglyceride content, lipogenesis-related gene expression and hepatic glucose uptake, and reduced fatty acid oxidation [8, 24, 36]. A role for C5L2 and ASP in liver regeneration in mice has also been suggested, as administration of ASP in C3KO mice restores adequate liver regeneration, an effect absent in C5L2 knockout mice [37]. Together, these findings suggest a protective role for C3/C3 peptides and C5L2 against the development of hepatic steatosis.

Based on this, we hypothesized that hepatic expression of C3, C3aR, C5aR, and C5L2 in humans would be associated with hormonal status and specific metabolic profiles in severely obese pre- and postmenopausal women. Further, we hypothesized that specific liver expression patterns might predict improvement in postoperative metabolic profile and liver enzymes of subjects over one year following biliopancreatic diversion surgery to induce weight loss.

## 2. Materials and Methods

*Ethics Statement.* The experimental protocol was approved by the university hospital ethics committee (CRIUCPQ: Centre de Recherche de l'Institut Universitaire de Cardiologie et Pneumologie du Québec) and all participants provided written informed consent for participation in medical research. 

*Study Subjects.* Samples were selected from the CRIUCPQ Tissue Bank (http://www.criucpq.ulaval.ca/index.php/en/tissue-bank) among severely obese women who had undergone bariatric surgery with biliopancreatic diversion (BPD) based on the following criteria: surgery within a 3-year period (2007–2010), no ovariectomy, no lipid lowering medication, no diabetes, and liver biopsy and blood sample availability. Of the 252 pre- and postmenopausal severely obese women identified, estradiol was measured (see below) and 91 subjects were chosen based on their plasma estradiol levels, <25th percentile (*n* = 46) and >75th percentile (*n* = 45) of the cohort tested. Fasting blood samples were collected prior to the surgery and at 3, 6, and 12 months postoperatively. Body composition was assessed prior to surgery and at 12 months. The experimental protocol was approved by the ethics committee of the CRIUCPQ and all participants provided written informed consent for participation in medical research. Patient selection criteria for bariatric surgery included body mass index (BMI), the presence of comorbidities, and a history of prior weight loss attempts. 

### 2.1. Clinical and Plasma Measurements

*Clinical Assessment.* Anthropometric measurements (height, weight, waist circumference, and hip circumference) were measured the day before surgery. Height, circumferences, and body weight of subjects were measured on a scale with 0.5 cm and 0.5 kg increments, respectively, and BMI (kg/m^2^) was calculated. Blood pressure was recorded using an automatic blood pressure cuff. 

*Preoperation Plasma Analysis.* Blood samples were obtained from participants after overnight fasting and collected into EDTA-containing tubes. The hospital clinical biochemistry laboratory measured fasting plasma glucose (GLU), triglyceride (TG), total cholesterol (TC), low density lipoprotein cholesterol (LDL-C), high density lipoprotein cholesterol (HDL-C), apolipoprotein B (Apo B), apolipoprotein A1 (Apo A1), and liver enzymes aspartate transaminase (AST), alanine transaminase (ALT), alkaline phosphatase (ALP), and gamma glutamyl transpeptidase (GGT). The following assays were measured directly in the research laboratory: Estrone (E1) and estradiol (E2) (both RIA, Beckman Coulter Canada LP, Mississauga, ON), adiponectin (RIA, Millipore, Billerica. MA, USA), and sex hormone binding globulin (SHBG) (ELISA, ALPCO, Salem, NH, USA) following instructions of the manufacturers. ASP concentration was measured using an in-house sandwich ELISA, following previously published methodology [38]. 

*Postoperation Analysis.* Weight, fasting TG, HDL-C, LDL-C, GLU, and liver enzymes (ALT, AST, ALP, and GGT) were measured at 3, 6, and 12 months after surgery as described above. 

### 2.2. Liver Sampling and Analysis

*Biopsies.* Liver biopsies were performed according to standard CRIUCPQ Tissue Bank procedures and approved by the ethics committee of the CRIUCPQ. Liver biopsy samples (250–500 mg tissue) were obtained during bariatric surgery. The liver biopsies were washed with sterile Kreb-Ringer-HEPES buffer, placed in liquid nitrogen, and then immediately transported and stored at −80°C. 

*RNA Extraction and Real-Time qPCR Analysis.* All samples (maximum 40 mg liver tissue) were homogenized in Qiazole (Qiagen Inc, Mississauga, ON, Canada). Total RNA was extracted from homogenates using the RNeasy Plus Universal Mini Kit (Qiagen Inc.) according to the manufacturer's instructions. From the total amount, 0.1 *μ*g of purified RNA was retrotranscribed to cDNA using a QuantiTec Reverse Transcription Kit (Qiagen Inc.) with a final volume of 20 uL. Genomic DNA contamination was eliminated by DNase treatment included in QuantiTec Reverse Transcription Kit. For real-time PCR evaluation of gene expression, 1 uL of cDNA was used for each reaction. RT2 SYBR Green qPCR Master Mix (Qiagen Inc.) was used and a 3-step PCR was performed using CFX96 Real-Time PCR Detection System (Bio-Rad Laboratories, Mississauga, ON, Canada), using the following protocol: an initial denaturation step at 95°C for 10 minutes, 40 cycles of 95°C for 10 s, 55°C for 10 s, and 72°C for 30 s were followed by a final extension step of 95°C for 10 s and melt curve 65°C to 95°C. Real-time RT-PCR was performed to quantify human C3, C3aR, C5aR, and C5L2 relative to glyceraldehyde-3-phosphate dehydrogenase (GAPDH) as a housekeeping gene, with primers obtained from Alpha-DNA (Montreal, Canada).

The sequences for the primers used were C5L2/GPR77-Right; 5′-TCCGAGAGGTCGCTGTAATC-3′, C5L2/GPR77-Left; 5′-TCAAGGACTCCCAAAACCAG-3′, C3aR1-Right; 5′-AGCAGAGAAAGACGCCATTG-3′, C3aR1-Left; 5′-ACTGTGGCTAAGTGTGGGGA-3′, C5R1-Right; 5′-TATCCACAGGGGTGTTGAGG-3′, C5R1-Left; 5′-GCCCAGGAGACCAGAACAT-3′, C3-Right; 5′-GCAATGATGTCCTCATCCAG-3′, C3-Left; 5′-CCTGGACTGCTGCAACTACA-3′, and GAPD-Right; 5′-AATGAAGGGGTCATTGATGG-3′, GAPD-Left; 5′-AAGGTGAAGGTCGGAGTCAA-3′. For data analysis the ΔΔCt method was used, as performed with Bio-Rad CFX manager software (version 1.5) (Bio-Rad Laboratories). 

*Statistics.* Statistical analysis was performed using GraphPad Prism 5 (GraphPad software, CA, USA) or Sigmastat 3.5 (Systat software, San Jose, CA, USA). Descriptive parameters are provided as mean ± SEM or in percentages. For nonnormally distributed parameters, values were log-transformed for statistical analysis. Two-tailed *t*-tests were used to analyze the differences between means and proportions of two groups. Two-way ANOVA analyses were used to compare among groups (as indicated). Pearson correlation was used to analyze bivariate correlation. Fisher exact test was used in the analysis of 2X2 contingency tables. Stepwise forward multiple regression analysis was used to assess how gene expression predicts variables of the metabolic profile. A *P* < 0.05 was considered statistically significant for all analyses.

## 3. Results

**Figure 1:**
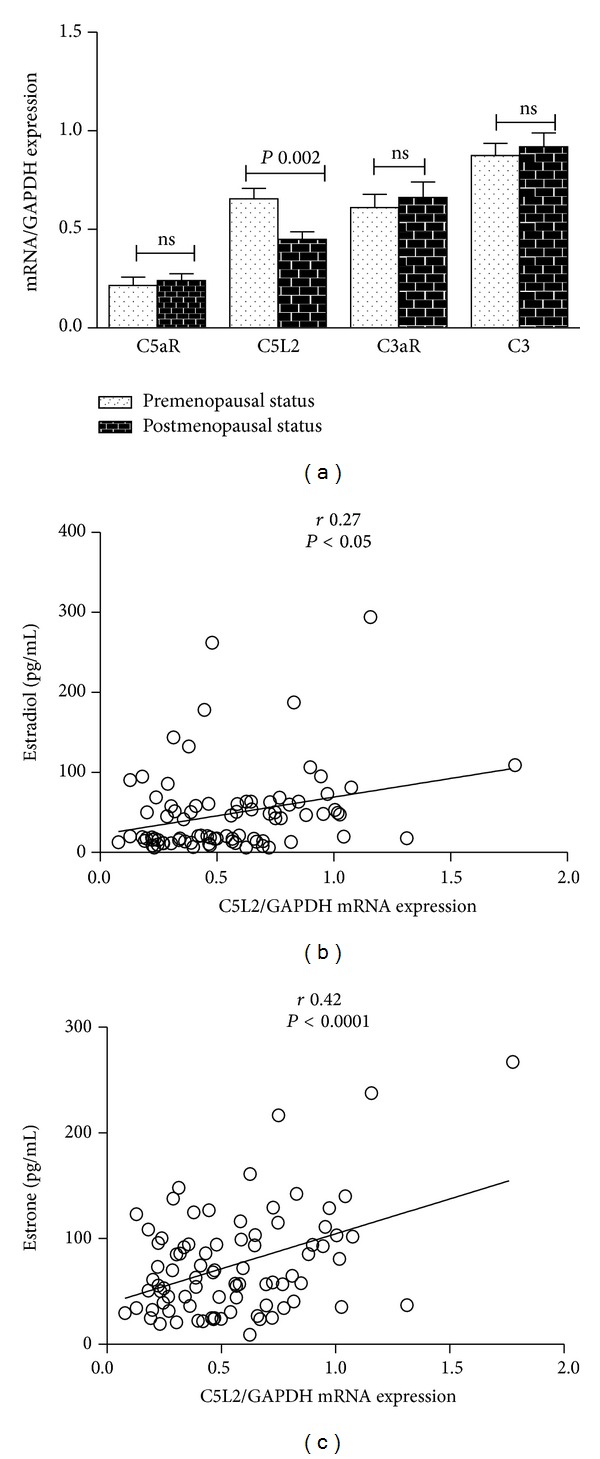
Association of liver C5L2 mRNA expression with circulating estrogen levels. mRNA was extracted from liver biopsies and reverse-transcribed to cDNA and gene expression was evaluated by RT-PCR with the indicated primers with all results expressed relative to GAPDH using the ΔΔCt method as described in detail in Section 2. (a) Hepatic mRNA expression levels of C3 and the three related receptors (C5L2, C3aR, and C5aR) in the pre- versus postmenopausal groups. ((b) and (c)) Linear correlation plot of C5L2 mRNA expression with plasma estradiol (E2) and estrone (E1).

*Relationship of Hormone Status with Gene Expression.* Baseline patient characteristics of the 91 severely obese women ranging in BMI (37.5 ≥ BMI ≥ 78.9 kg/m^2^) and age (21–69 years) are given in Table 1. The subjects were grouped, based on fasting estradiol (E2), into pre- and postmenopausal status groups as shown in Table 1. Biochemical analysis indicated that subjects in the high E2 subgroup (*P* < 0.0001) also had higher levels of estrone (E1) (*P* < 0.0001) and progesterone (*P* < 0.05) and were younger (*P* < 0.0001) reflecting their premenopausal status. The high E2 group also had lower Apo B levels (*P* < 0.0001), higher Apo A1 (*P* = 0.025), and higher ApoA1/B ratio (*P* < 0.0001), despite having significantly higher waist circumference (*P* < 0.05) and lower HDL-C (*P* < 0.05). Hepatic gene expression of C3 and the three related receptors (C5L2, C3aR, and C5aR) in pre- versus postmenopausal groups are shown in Figure 1(a). While expression levels of C3, C3aR and C5aR were not different between the two groups, C5L2 gene expression was higher in premenopausal status women (higher E2) (*P* < 0.01). Further, C5L2 gene expression also correlated directly with plasma E2 and E1 levels (E2 *r* = 0.27, *P* < 0.05; E1 *r* = 0.42, *P* < 0.0001) as shown in Figures 1(b) and 1(c), while C3 correlated inversely with SHBG (*r* = −0.28, *P* < 0.01, data not shown). 

**Figure 2:**
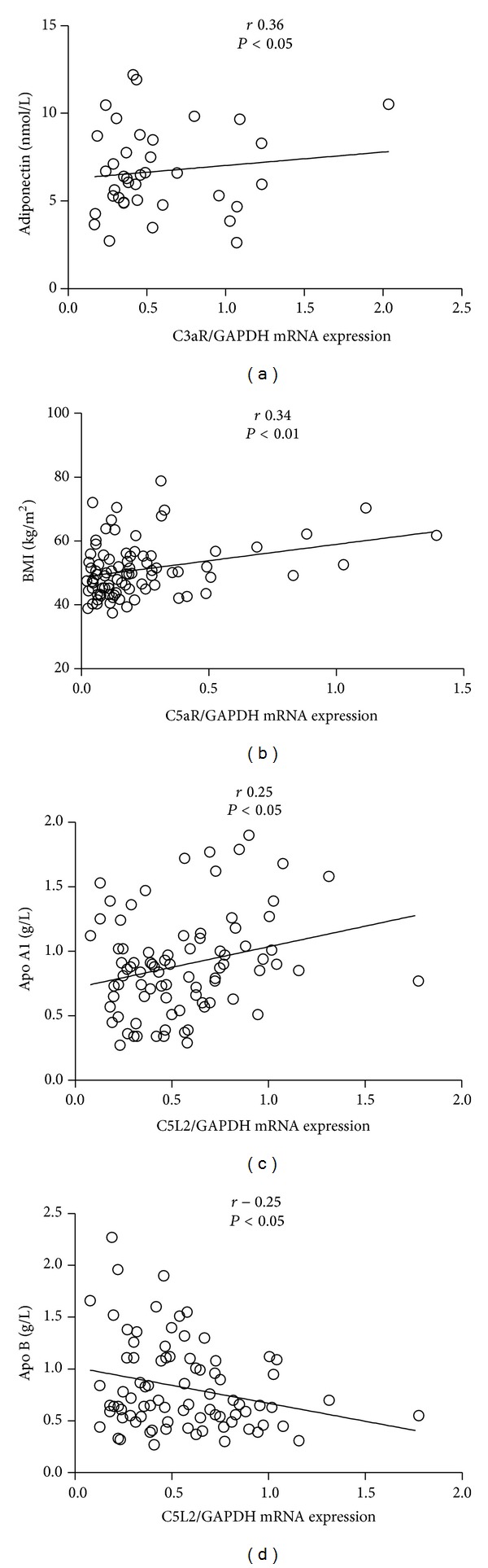
Hepatic mRNA Expression relative to metabolic parameters. (a)–(d): Linear plot correlations of hepatic mRNA expressions and metabolic parameters. mRNA was extracted from liver biopsies and reverse-transcribed to cDNA and gene expression was evaluated by RT-PCR with the indicated primers with all results expressed relative to GAPDH using the ΔΔCt method as described in detail in Section 2.

*Hepatic Complement Gene Expression and Metabolic Profile.* The relationships between hepatic gene expression and various anthropometric parameters and metabolic profile were then evaluated. Hepatic C3 expression correlated inversely with the liver enzyme GGT (*r* = −0.28, *P* < 0.05, data not shown). C3aR was associated with adiponectin (Figure 2(a), *r* = 0.36, *P* < 0.05) while C5aR correlated with BMI (Figure 2(b), *r* = 0.34, *P* < 0.01) and liver enzyme AST (*r* = 0.32, *P* < 0.05, data not shown). Hepatic gene expression of C5L2 correlated with ApoA1 (Figure 2(c), *r* = 0.25, *P* < 0.05), ApoA1/B (*r* = 0.25, *P* < 0.05, data not shown), and waist circumference (*r* = 0.25, *P* < 0.05, data not shown), with inverse correlations between C5L2 and ApoB (Figure 2(d), *r* = −0.25, *P* < 0.05), age (*r* = −0.26, *P* < 0.05, data not shown), and LDL-C (*r* = −0.23, *P* < 0.05, data not shown).

Forward stepwise regression analyses indicated that the dependent variable C5aR could be predicted from a linear combination of the independent variables age, BMI, and glucose (pooled *r* ^2^ = 0.20, *P* < 0.001) and the dependent variable C5L2 could be best predicted from a linear combination of independent variables LDL-C and E1 (pooled *r* ^2^ = 0.24  *P* < 0.001). 

**Figure 3:**
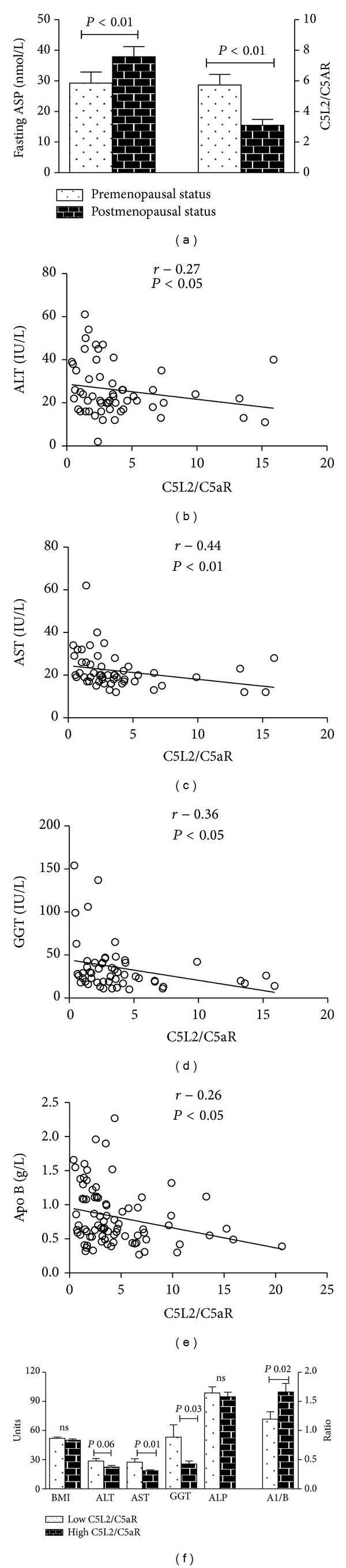
Association of hepatic C5L2/C5aR mRNA ratio with menopausal status and metabolic profile. mRNA was extracted from liver biopsies and reverse-transcribed to cDNA and gene expression was evaluated by RT-PCR with the indicated primers with all results expressed relative to GAPDH using the ΔΔCt method as described in detail in Section 2. (a) Comparison of hepatic C5L2/C5aR mRNA expression ratio and plasma ASP levels in premenopausal and postmenopausal groups. ASP values were log transformed for statistical analysis. ((b)–(d)) Linear plot correlation of liver enzymes ALT, AST, and GGT with C5L2/C5aR mRNA ratio. (e) Linear plot correlation of ApoB and C5L2/C5aR mRNA ratio. (f) Comparison of preoperative BMI (kg/m^2^) and fasting liver enzymes (IU/L) (left *y*-axis) and ApoA1/B ratio (right *y*-axis) before bariatric surgery in subjects separated based on low versus high C5L2/C5aR mRNA ratio, compared by unpaired Student's *t*-test.

*C5L2/C5aR Ratio and Metabolic Parameters.* As recent* in vitro* experimental evidence indicates a direct interaction (heterodimerization) between C5aR and C5L2 receptors [26, 39] which could affect subsequent cellular signaling and response, we tested associations of the C5L2/C5aR mRNA ratio with various metabolic indices in our subjects. While plasma ASP levels were significantly lower in premenopausal women versus postmenopausal women (*P* < 0.01), the hepatic C5L2/C5aR mRNA ratio was higher (Figure 3(a), *P* < 0.001). Further, positive correlations between C5L2/C5aR mRNA ratio and sex hormones were identified (E1: *r* = 0.28, *P* < 0.01 and E2: *r* = 0.38, *P* < 0.001, data not shown). Interestingly, the hepatic C5L2/C5aR mRNA ratio was inversely associated with circulating liver enzymes (ALT, AST, and GGT) and ApoB (Figures 3(b)–3(e)), as well as age (*r* = −0.29, *P* < 0.01, data not shown). Because of significant correlations between the C5L2/C5aR mRNA ratio and preoperative liver enzymes and metabolic parameters, subjects were partitioned based on high versus low C5L2/C5aR mRNA ratio. Before bariatric surgery, ALT, AST, and GGT but not ALP were significantly higher in the low-C5L2/C5aR group, with a lower ApoA1/B ratio, although there was no significant difference in BMI between these two groups (Figure 3(f)). 

**Figure 4:**
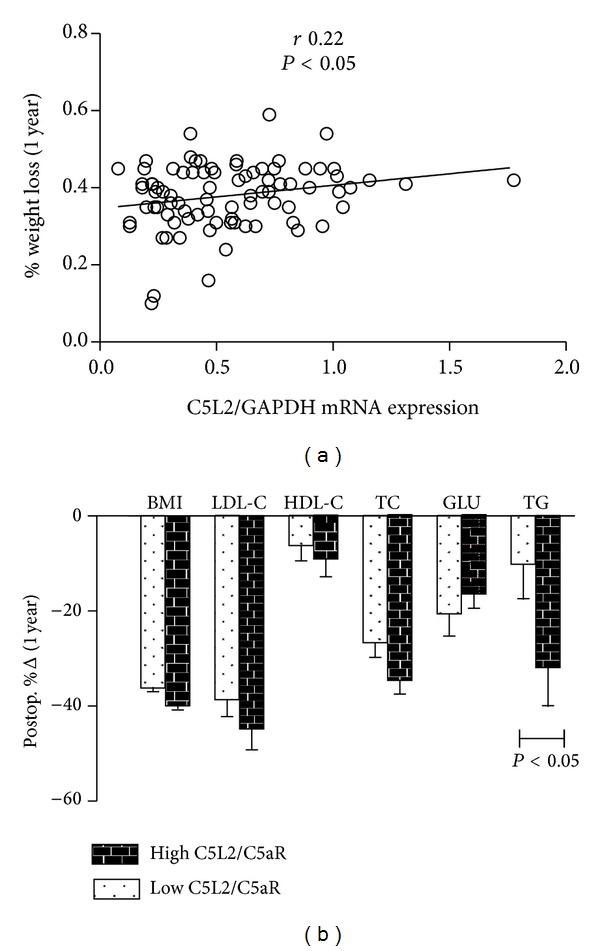
Metabolic profile after surgery. mRNA was extracted from liver biopsies and reverse-transcribed to cDNA and gene expression was evaluated by RT-PCR with the indicated primers with all results expressed relative to GAPDH using the ΔΔCt method as described in detail in Section 2. (a) Linear correlation plot of liver C5L2 mRNA expression at time of surgery versus % weight loss 12 months after surgery, analyzed by Pearson correlation test. (b) Percentage changes of metabolic profile before and after bariatric surgery and categorical comparisons between the high and low C5L2/C5aR mRNA ratio subgroups, analyzed by Fisher exact test. BMI: body mass index; GLU: fasting glucose; HDL-C: high density lipoprotein-cholesterol; LDL-C: low density lipoprotein-cholesterol; TG: triglyceride; TC: total cholesterol.

**Figure 5:**
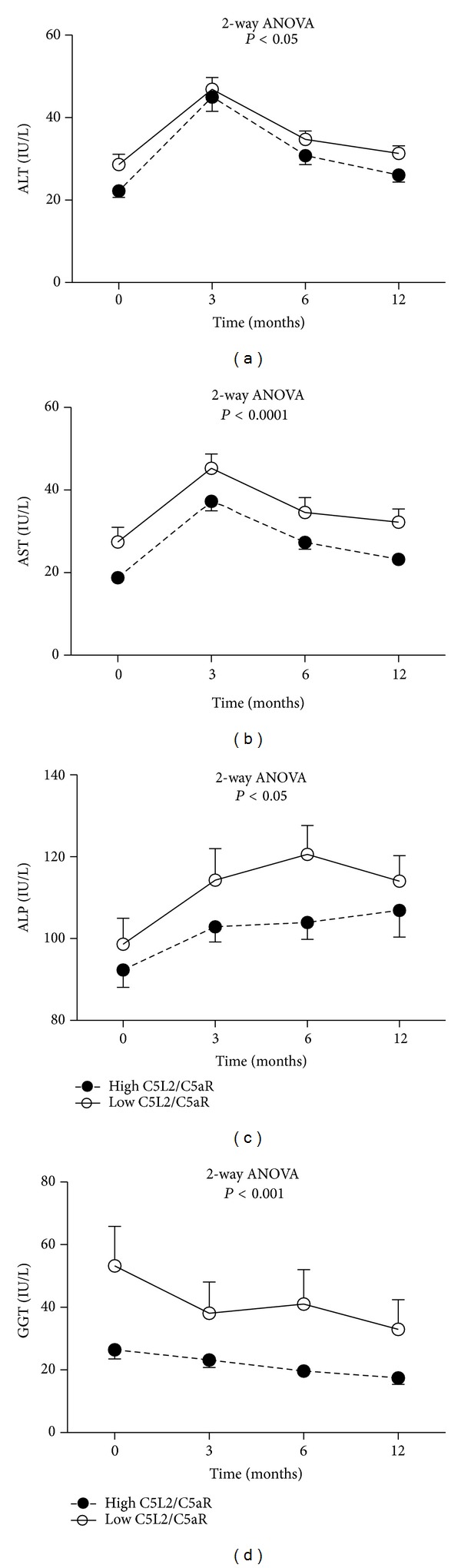
Plasma liver enzyme levels relative to C5L2/C5aR Expression. ((a)–(d)) Plasma liver enzyme levels before surgery and during one year following surgery in high versus low C5L2/C5aR mRNA ratio subgroups, 2-way ANOVA comparison.

*Relationship of C5L2/C5aR Ratio with Bariatric Surgery Outcome.* Metabolic profile and anthropometric results were evaluated postoperatively and compared to baseline data. As expected, the anthropometric and metabolic profile of subjects substantially improved over a 12-month follow-up after bariatric surgery. Hepatic C5L2 mRNA expression was positively associated with % weight loss (Figure 4(a), *r* = 0.22, *P* < 0.05). Following bariatric surgery, subjects were routinely followed in the surgical clinic at 3, 6, and 12 months. Globally, over the 12-month period, all liver enzyme levels in the low C5L2/C5aR mRNA ratio group remained higher than in the high C5L2/C5aR mRNA ratio group (Figures 5(a)–5(d), ALP *P* < 0.05, ALT *P* < 0.05, AST *P* < 0.001 and GGT *P* = 0.0002 by 2-way ANOVA), although there was no significant difference in BMI reduction curves (data not shown). One-year changes in BMI, LDL-C, HDL-C, total cholesterol, glucose, and triglyceride are shown in Figure 4(b). Although the expected improvements in all parameters were not significantly different between the two groups, the relative change in TG was significantly different between the low and high C5L2/C5aR mRNA groups (Fisher exact test, *P* = 0.02).

## 4. Discussion

In this study we investigated liver tissue mRNA expression of complement C3 and its cleavage product receptors, C3aR, C5aR, and C5L2 and their relationship with hormonal status among severely obese women who underwent BPD. In addition, we assessed the subjects' metabolic profile before and up to one year after surgery in relation to partitioning between high and low liver C5L2/C5aR mRNA ratio. The major findings of this study are (1) associations of hepatic complement-related gene expression in severely obese women with sex hormones and metabolic profile and (2) associations of hepatic C5L2/C5aR ratio with pre- and postoperative metabolic profile.

Although many studies have examined the role of complement C3, and to a lesser extent, the role of related receptors C5L2, C5aR, and C3aR, overall the major focus of these studies has been in an immune context. More recently, however, the impact of C5L2, C5aR, and C3aR in the area of energy (lipid and glucose) metabolism has been recognized as a result of studies in gene knockout mice [8–10, 14].

However, there is little information available on gender and sex hormone influence on complement-related receptors and their potential roles in liver function. In the present study, the positive correlation of hepatic C5L2 (and C5L2/C5aR ratio) with circulating estrogens (E1, E2) and the decrease in expression in postmenopausal women indirectly suggest an estrogenic influence.* In vitro* cellular and* in vivo* animal studies support the notion that estrogens increase adipose C3 expression [31] and neural C5aR expression [32] but have differential effects on C5L2 depending on estrogen receptor targeting (ER*α* and *β*) in different tissues [33]. It is now well recognized that most tissues in both men and women are influenced by estrogens [40], and the liver is particularly sensitive, including effects on lipid metabolism [41]. Estrogen withdrawal results in increased hepatic lipogenesis, decreased VLDL lipoprotein production and secretion, and decreased lipid oxidation with associated gene expression changes [41, 42]. In the present study, given the correlations between C5L2 and various lipid parameters, including apoA1 and apoB, estrogens may mediate the effects either directly or indirectly via hepatic lipid changes.

The increased plasma ASP levels commonly seen in metabolic dysfunction (including type 2 diabetes and cardiovascular diseases) have been proposed as indicating an “ASP resistant state” [14, 24, 43]. A recent study demonstrated that diet-induced obesity in mice results in increased plasma ASP, decreased adipose C5L2 expression, and decreased* in vivo* ASP response, providing proof-of-concept of ASP resistance [44]. The lower hepatic C5L2/C5aR mRNA ratio with yet higher ASP plasma level in postmenopausal obese women suggests the presence of ASP resistance in hepatic cells. Pathologically, if the ASP/C5L2 signaling is disturbed in adipose tissue, as in the case of decreased expression of the ASP receptor (a decrease in ASP function), this may enhance diversion of available glucose and fatty acids to other tissues (muscle, liver, and arterial wall), leading to lipotoxicity, unless disposal mechanisms are upregulated such as increased fatty acid oxidation [45]. This consequence was demonstrated in C5L2KO mice where it was shown that, in response to reduced TG storage in white adipose tissue, C5L2KO mice developed a compensatory mechanism of increased muscle fat oxidation [23]. We speculate that the presence of ASP resistance in postmenopausal women may contribute to their metabolic dysfunction.

The C5L2/C5aR mRNA ratio was also informative in predicting postoperative outcomes: higher values of this ratio were associated with greater relative weight loss, greater decreases in fasting TG, and lower liver enzymes both before surgery and over the follow-up. In the preoperative state, increased C5L2/C5aR mRNA ratio was associated with lower BMI, apoB, and liver enzymes, and yet higher apoA1. Overall, these data suggests that a high hepatic C5L2/C5aR mRNA ratio is a beneficial feature and raises the question of what the specific functional roles of C5L2 and C5aR are in relation to lipid metabolism in the liver.

While there is little data on the function of complement-related receptors directly in liver cells (either parenchymal hepatocytes or nonparenchymal cells), the complement system may contribute in either a beneficial or detrimental manner. In cells, C3 production in hepatocytes is stimulated by PPAR*α*, TNF*α*, IL-6, and LPS [46, 47] while C5a, via interaction with hepatic C5aR, is involved in proliferation, glycogen phosphorylase, and glucose output [48–50].* In vivo*, the complement system can both promote inflammation/injury and play a homeostatic role in repairing damaged tissue. While C3, C3aR, C5aR, and C5L2 blockade/deficiency can protect from liver failure and improve sepsis survival in mice [51, 52], by contrast, the presence of these same proteins, as well as the C3 products ASP and C3a, plays roles in promoting liver regeneration, liver transplant tolerance, and protection from steatosis [9, 37, 53, 54].

In relation to metabolic effects, an obesogenic diet induces increased liver expression of C3, C5aR, and C5L2 [8, 14, 55], while C3KO, C5aRKO, C5L2KO, and C3aRKO mice are resistant to diet-induced obesity [8, 9, 23, 24, 56]. Finally, C3 and ASP/C3a have been implicated in human studies of fatty liver disease [57, 58].

How the balance between the various ligands (C3a, ASP, and C5a/C5adesArg) and their receptors (C3aR, C5L2, and C5aR) could influence overall hepatic function remains to be elucidated. Recent studies suggest that the ability of several ligands binding to the same receptor to evoke differential signaling and biological responses constitutes a phenomenon referred to as “biased agonism” [59]. Depending on the local environment and the circumstances under which complement proteins are generated, they may contribute to cell and tissue homeostasis, to benefit or burden inflammation, or to promote tissue regeneration versus fibrosis [7]. For example, while in mid-grade sepsis, blockage (or gene absence) of either C5aR or C5L2 improved survival, in high-grade sepsis combined blockage of these two receptors was necessary [19]. Further, ASP demonstrated a biphasic role with a balance between inflammation/injury versus regeneration [37].

While C3aR neither homodimerizes nor heterodimerizes, by contrast, C5aR and C5L2 both homo- and heterodimerize [26, 60]. The potential for cooperative interaction between C5L2 and C5aR has been evaluated in* in vivo* and* in vitro* studies. C5L2 acts as a positive modulator for both C5a- and C3a-induced responses in neutrophils, macrophages, and fibroblasts and is critical for optimal signaling [61]. Conversely, another study on human neutrophils demonstrated that C5L2 functions as an intracellular receptor, becoming colocalized with C5aR after C5a binding, acting as a negative modulator through the beta-arrestin pathway [39]. Further, a recent study demonstrated formation of C5L2/C5aR heterodimers in adipocytes and macrophages, with cointernalization/colocalization upon stimulation with either ASP/C3adesArg or C5a [26]. Such dimerization may be one mechanism underlying the potential positive/negative modulatory effect of C5L2 on C5a or ASP/C3a effector functions.

Limitations of this study should be noted: all of the interpretations and conclusions are based on the evaluation of obese subjects, and all analyses were in women. In addition, based on the limited availability of small quantities of frozen tissue from liver biopsies, the present study relied on C5aR and C5L2 mRNA expression without addressing the possible posttranslational modifications, protein levels, or functional assays of the examined receptors. Further, as this is a cross-sectional study, the potential links between cause and effect can only be speculated upon.

## 5. Conclusion

In this study, evidence is presented to integrate data suggesting that complement-related proteins correlate with sex hormones and metabolic profile in liver tissue. C5L2-C5aR interactions and the association with other mediators, such as estrogens, may have a role in metabolic and inflammatory functions of complement-related proteins in human liver cells.

## Figures and Tables

**Table 1:** Characteristics of pre- and postmenopausal Obese Women.

Parameters	All subjects (pooled)			Premenopausal status	Postmenopausal status	*t*-test
	Mean (*n* = 91)	Min	Max	Mean (*n* = 45)	Mean (*n* = 46)	
Estradiol (pg/mL)	47.6 ± 5.3	6.1	294	81.4 ± 8.2	14.6 ± 0.7	N/A
Age (yrs)	40 ± 1	21	69	35 ± 1	45 ± 2	*P* < 0.01
BMI (kg/m^2^)	51.1 ± 0.9	37.5	78.9	51.9 ± 1.4	50.3 ± 1.1	NS
Waist circumference (cm)	146 ± 2	119	184	150 ± 2	142 ± 2	*P* < 0.05
Hip circumference (cm)	137 ± 2	97	177	136 ± 3	138 ± 2	NS
SBP (mmHg)	136 ± 2	109	186	137 ± 2	136 ± 2	NS
DBP (mmHg)	84.4 ± 1.3	43.0	113	83.3 ± 2.2	85.5 ± 1.4	NS
Glucose (mmol/L)	5.6 ± 0.1	3.7	11.6	5.4 ± 0.2	5.7 ± 0.1	NS
Cholesterol (mmol/L)	4.9 ± 0.1	3.1	7.3	4.8 ± 0.1	5.0 ± 0.1	NS
HDL-C (mmol/L)	1.31 ± 0.03	0.61	2.26	1.24 ± 0.04	1.37 ± 0.05	*P* < 0.05
LDL-C (mmol/L)	2.8 ± 0.1	1.0	4.9	2.8 ± 0.1	2.8 ± 0.1	NS
Triglyceride (mmol/L)	1.7 ± 0.1	0.6	4.5	1.7 ± 0.1	1.8 ± 0.1	NS
Apo B (g/L)	0.84 ± 0.05	0.27	2.27	0.64 ± 0.04	1.04 ± 0.07	*P* < 0.001
Apo A1 (g/L)	0.81 ± 0.04	0.06	1.95	0.98 ± 0.04	0.79 ± 0.07	*P* < 0.05
Apo A1/B	1.4 ± 0.1	0.1	4.6	1.8 ± 0.1	1.1 ± 0.1	*P* < 0.001
Adiponectin (ug/mL)	7.5 ± 0.9	2.5	41.1	6.3 ± 0.5	9.6 ± 1.9	NS
Estrone (pg/mL)	75 ± 5	9	267	106 ± 7	44 ± 4	*P* < 0001
SHBG (pg/mL)	181 ± 12	15.2	573	162 ± 15	199 ± 18	NS
Progesterone (ng/mL)	2.4 ± 0.3	0.1	6.9	2.9 ± 0.4	1.6 ± 0.3	*P* < 0.05
AST (IU/L)	23.4 ± 1.9	12.2	123	20.5 ± 6.6	25.1 ± 18.9	NS
GGT (IU/L)	40.3 ± 6.8	10.1	382	39.1 ± 75.2	40.9 ± 33.7	NS
ALP (IU/L)	95.5 ± 3.8	49.0	210	99.9 ± 28.8	92.7 ± 29.8	NS
ALT (IU/L)	25.5 ± 1.5	2.0	61.0	23.8 ± 11.3	26.6 ± 12.6	NS

Estradiol was measured in the 252 severely obese women evaluated, and subjects in the lowest quartile (<25th percentile, *n* = 46 postmenopausal) and highest quartile (>75th percentile, *n* = 45 premenopausal) were selected for further evaluation of plasma and liver tissue and were compared by *t*-test. Apo: apolipoprotein; ALT: alanine transaminase; ALP: alkaline phosphatase; AST: aspartate transaminase; BMI: body mass index; DBP: diastolic blood pressure; GGT: gamma-glutamyl transpeptidase; HDL-C: high density lipoprotein-cholesterol; LDL-C: low density lipoprotein-cholesterol; SHBG: sex hormone-binding globulin; SBP: systolic blood pressure. *P* < 0.05 is considered significant.

## Abbreviations

ALT: Alanine transaminase
ALP: Alkaline phosphatase
ApoA1/B: Apolipoprotein A1/B
ASP: Acylation stimulating protein
AST: Aspartate transaminase
BPD: Biliopancreatic diversion
BMI: Body mass index
C5L2: C5a-like receptor 2
DBP: Diastolic blood pressure
E1: Estrone
E2: Estradiol
HDL-C: HDL cholesterol
GGT: Gamma glutamyl transpeptidase
GLU: Glucose
LDL-C: LDL cholesterol
SHBG: Sex hormone-binding globulin
SBP: Systolic blood pressure
TC: Total cholesterol
TG: Triglycerides.
